# Physical fitness and lifestyles associated with biological aging

**DOI:** 10.18632/aging.206031

**Published:** 2024-07-19

**Authors:** Takuji Kawamura, Radak Zsolt, Mitsuru Higuchi, Kumpei Tanisawa

**Affiliations:** 1 Faculty of Sport Sciences, Waseda Universitty, Saitama 359-1192, Japan; 2 Research Center for Molecular Exercise Science, Hungarian University of Sports Science, Budapest 1123, Hungary

**Keywords:** DNA methylation, epigenetic clock, physical fitness, anthropometry, blood biochemical parameters, nutritional intake

Given the growing aging population worldwide, it is now crucial to establish interventional strategies capable of targeting aging rather than organ- and disease-based segmented medicine [1]. The geroscience hypothesis, which proposes that delaying aging can prevent the onset of diseases [2], is rapidly expanding owing to improvements in the accuracy of aging biomarkers resulting from advances in both measurement (e.g., omics) and analytical (e.g., bioinformatics) technologies. Among these, DNA methylation-based aging clocks (DNAm aging clocks), which are calculated from age-related DNA methylation changes, are promising biomarkers that can predict “biological age” and an individual’s biological state with high accuracy. Therefore, geroprotectors targeting this novel biomarker of aging need to be developed. In this context, exercise is a powerful “geroprotector” that is well-recognized to extend the human health span. However, the relationship between physical fitness and biological age, based on the DNAm aging clock, is poorly understood. Most previous studies investigated the relationship between physical activity and DNAm aging clocks based on questionnaires and accelerometers using a molecular epidemiological approach [3]. Theoretically, physical activity and fitness differ, with physical fitness considered the result of exercise, which is planned and regular physical activity [4]. Considering that various health outcomes can be strongly associated with fitness, especially cardiorespiratory fitness (CRF), rather than with physical activity [5], CRF can be a stronger geroprotector than physical activity. Therefore, in the field of geroscience, it can be valuable to investigate the relationship between CRF and the DNAm aging clock and determine fitness reference values for delaying aging.

Here, we discuss our recent report on the “Associations between cardiorespiratory fitness and lifestyle-related factors with DNA methylation-based ageing clocks in older men: WASEDA’S Health Study” [6]. In this study, lifestyle-related factors such as anthropometric variables, blood biochemical parameters, nutritional intake status, smoking, alcohol intake, chronotype, and CRF, a promising predictor of all-cause mortality, were measured in 144 males aged ≥65 years. Using these measurements, we attempted to determine the relationship between CRF and various lifestyle-related factors associated with biological aging based on DNAm aging clocks. We found that CRF was negatively related to epigenetic age acceleration, even after adjusting for confounders (chronological age, smoking, and alcohol consumption), and that maintenance of CRF above a reference value (i.e., 22.7 mL/kg/min) was associated with lower age acceleration. Moreover, the maintenance of optimal body composition, adequate intake of carbohydrates and micronutrients, and a morning-type chronotype were associated with lower age acceleration. Conversely, excessive visceral fat accumulation, smoking, excessive alcohol consumption, and dyslipidemia were associated with acceleration of older age. Finally, the relative contribution of each lifestyle-related variable to age acceleration was suggested to be higher for calf circumference, serum triglycerides, carbohydrate intake, and smoking status than for CRF. Collectively, these findings suggest that although the relative contribution of CRF to biological aging is relatively low when compared with lifestyle-related variables, such as smoking, the maintenance of CRF is associated with delayed biological aging in older males.

Our findings have a manifold significance in the field of geroscience. Our study reinforces the geroscience concept that active lifestyle choices may impact quantifiable molecular biomarkers that capture biological aging. This is consistent with the concept of sports and health science, which aims to achieve independent living in later life through exercise and lifestyles that can be practiced by almost all individuals without depending on medical care or drugs and also encourages increased individual health awareness. Furthermore, this study provides a springboard for developing more effective lifestyle interventions. Based on our results, interventional strategies that preferentially target lifestyle variables with a high relative contribution to biological aging can be developed. On the other hand, our study has several limitations. First, it included only older male subjects; this demographic specificity implies that the results may not be applicable to a broader population. Second, the study did not account for all possible confounders that may influence the relationships between CRF, lifestyle-related factors, and biological aging, such as genetic predisposition and socioeconomic status. Therefore, future studies are needed to consider sex, ethnicity, genetic predisposition, and socioeconomic status to demonstrate the independent relationships between CRF, lifestyle-related factors, and biological aging.

In addition to these limitations regarding population characteristics and confounders, the central concern of exercise scientists is determining the causal relationship between CRF and biological aging. Several cross-sectional studies, including ours, have only demonstrated the relationship between physical activity, physical fitness, and biological aging [3, 6, 7]; however, the causal relationship between them remains elusive. To prove a causal relationship, it is necessary to conduct longitudinal studies that track age-related changes in both CRF and DNAm aging clocks, as well as endurance exercise training intervention studies. In mouse studies, late-life exercise training could delay epigenetic aging of skeletal muscle [8]. In humans, exercise training reportedly leads to epigenetic patterns toward a younger profile [9]. Another study has reported that the number of subjects with higher baseline levels of epigenetic age acceleration decreased after exercise training [10]. These findings suggest that exercise training has beneficial effects on biological aging. Future investigations are needed to clarify the effects of different training variables such as intensity, time, frequency, duration, and modality of exercise on biological aging. In addition, it is important to mention that exercise has a systemic health-promoting effect [11], raising the essential question of whether exercise consistently delays the biological aging of multiple organs. If this research question is answered, we can gain a better understanding of the mechanisms underlying the systemic effects of exercise.

Exercise-related geroscience research is currently in its infancy; thus, a steady accumulation of evidence is required. Assuming that regular exercise has a beneficial effect on biological aging, in the future, individuals may regularly assess biological aging in addition to other health outcomes such as body weight, muscle mass, and limb and waist circumferences. However, it should be noted that exercise is not a panacea, and continued research without bias is crucial. In fact, it should be emphasized that our study found that the relative contribution of CRF to biological aging was relatively low when compared with that of other lifestyle-related factors, such as smoking. Finally, it should be highlighted that the DNAm aging clock is not a perfect aging biomarker, and whether it is a cause or a consequence of aging remains debatable. Therefore, it would be useful to examine not only the DNAm aging clocks developed from specific CpG sites but also global DNA methylation and promoter methylation of individual genes, which would lead to a better understanding of the geroprotective effects of exercise.
